# Right Hepatic Artery Forming Moynihan’s Hump in the Calot’s Triangle: An Unwanted Finding During Laparoscopic Cholecystectomy

**DOI:** 10.7759/cureus.97397

**Published:** 2025-11-21

**Authors:** Dimosthenis Chrysikos, Nikolaos Taprantzis, Panagiotis Kanavaros, Amir Shihada, Theodore Troupis

**Affiliations:** 1 Department of Anatomy, National and Kapodistrian University of Athens School of Medicine, Athens, GRC; 2 Department of Anatomy-Histology-Embryology, University of Ioannina, Ioannina, GRC

**Keywords:** biliary surgery, calot’s triangle, caterpillar’s hump, moynihan’s hump, vascular malformation

## Abstract

The term Moynihan’s hump or Caterpillar’s hump is used to describe a “twisting” or “tortuous” right hepatic artery inside Calot’s triangle. This abnormal artery passes in proximity to the gallbladder or cystic duct, posing a serious potential danger for surgeons operating on that area. In this case report, we present a patient who underwent a laparoscopic cholecystectomy due to gallstones within the gallbladder. During the surgical procedure, a tortuous right hepatic artery was identified within Calot’s triangle, running adjacent to the gallbladder and cystic duct. Clipping and division of the short cystic artery allowed the artery to be mobilized safely, and the gallbladder was subsequently detached from the liver bed. The patient tolerated the whole procedure well, and his postoperative course was complication-free. Good anatomical knowledge is needed to identify the abnormality and prevent serious injury.

## Introduction

In general, the hepatocystic triangle, also known as Calot’s triangle (described by Jean-Francois Calot [1]), is a physiological space of triangular formation that is observed in the transverse fissure of the liver. Its significance is mainly surgical, as it is dissected during a cholecystectomy procedure [2]. Its anatomical contents consist of the right hepatic artery, cystic artery, cystic lymph node, connective tissue, lymphatics, and accessory hepatic ducts and arteries. The inferior boundaries of Calot’s triangle are determined by the presence of the cystic duct, which passes downwards and along the left side of the gallbladder. The left boundary is defined by the common hepatic duct, while the superior boundary is set by the inferior surface of the liver [2,3]. It should be noted that the cystic artery, as well as the cystic duct, holds great importance, as they should be identified before ligation to avoid serious unwanted injury [3]. In other words, a detailed understanding and thorough anatomical knowledge of the area of interest is required to prevent any iatrogenic injury. Among the many anatomical variations that have been reported in Calot’s triangle, this case report will focus on a surgically significant feature of the right hepatic artery, called “Moynihan’s hump” [3]. In this unique formation, the right hepatic artery can follow a “twisting” path inside the hepatocystic triangle and come in proximity with the gallbladder or the cystic duct. Following this course, the right hepatic artery has been noted to branch into a cystic artery of unusually short length [4]. In this anomaly, the right hepatic artery can be mistaken for the cystic artery, making it prone to injury during surgical removal of the gallbladder, whether laparoscopic or open. According to a systematic review by Asghar et al., which included over 8,000 subjects, Moynihan’s hump was present only in 3.81% of the total study population. Moreover, the frequency was 7.22% in cadaveric data, while only 3.1% in surgical sites [5]. Other sources argue that the frequency of such an abnormal formation is reported to be from 1.3% to 13.3% [6].

## Case presentation

A 55-year-old Caucasian male presented with atypical symptoms of upper abdomen pain. The patient’s health history was characterized as unremarkable. After clinical examination, mild tenderness in the epigastrium and the upper right quadrant was noted. No abnormalities were detected in the laboratory examinations. An abdominal ultrasound (US) was ordered, which depicted a gallbladder containing stones with a maximum diameter of 2 cm. His past surgical history included an appendicectomy in childhood. No further examination was ordered, and the patient was subjected to a laparoscopic cholecystectomy.

Surgical procedure

**Figure 1:**
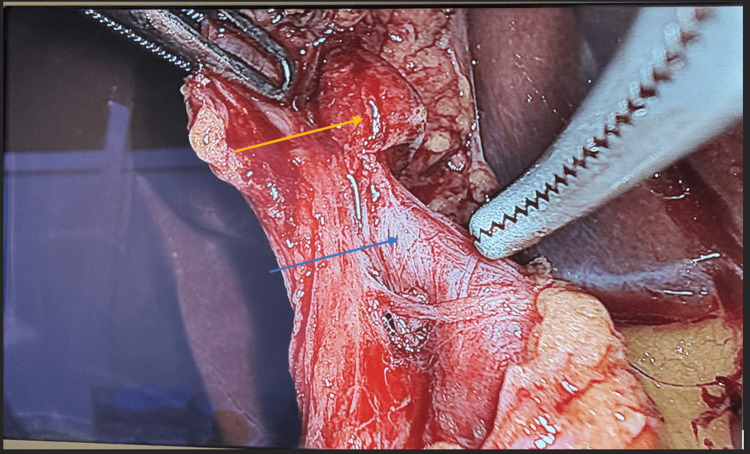
Intraoperative view of Calot’s triangle. The blue arrow depicts an unexpected finding entering Calot’s triangle, proven to be Moynihan’s hump. The yellow arrow depicts an enlarged Calot’s lymph node.

**Figure 2:**
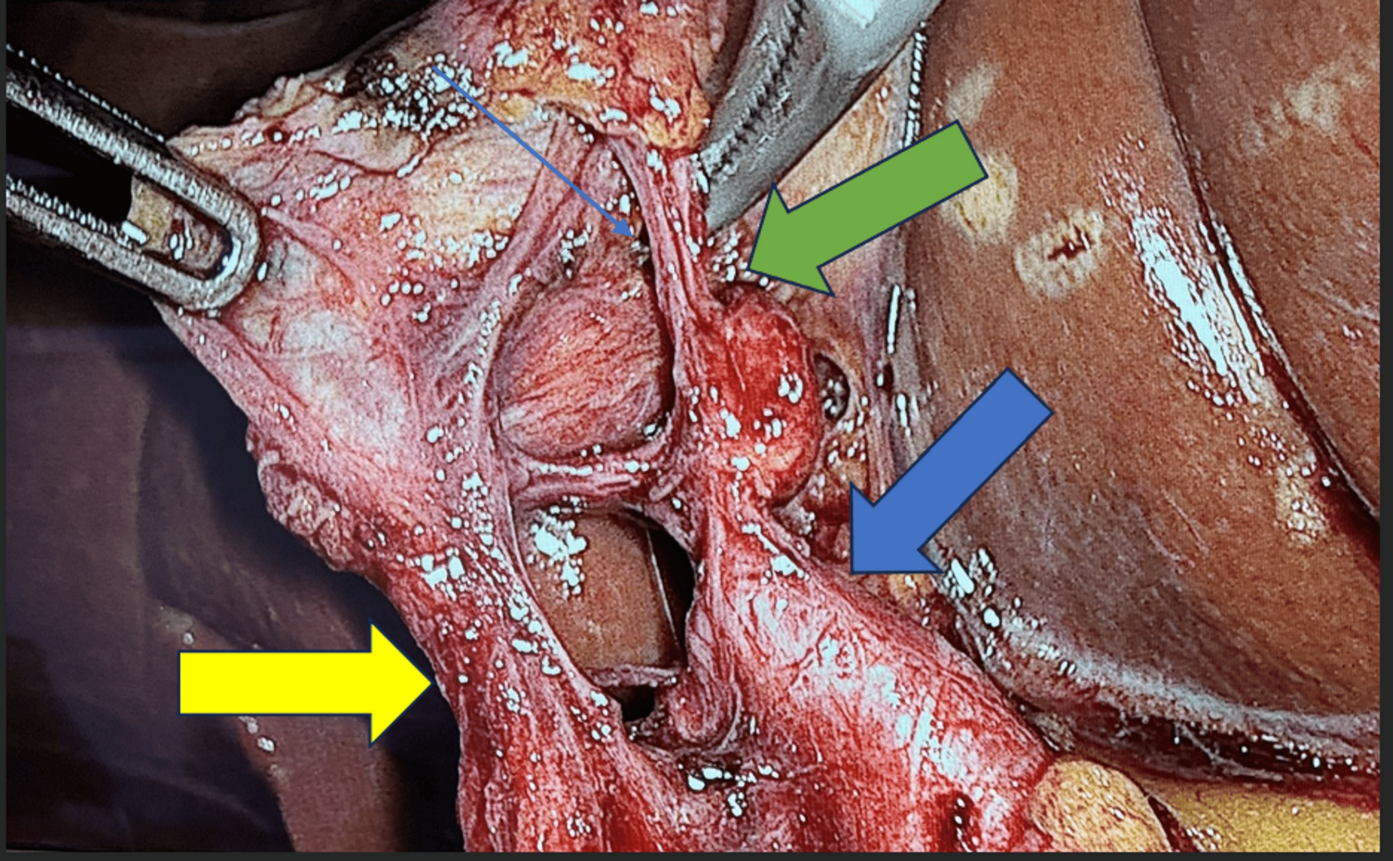
Intraoperative view of the critical vessels. The big blue arrow depicts the right hepatic artery, the small blue arrow depicts Moynihan’s hump, the green arrow depicts the cystic artery, and the yellow arrow depicts the cystic duct.

The patient was positioned in the reverse Trendelenburg with left lateral position under general anesthesia. After establishing a 14 mmHg pneumoperitoneum using a Chason open technique, a four-port technique was used with a 30° camera through a 12-mm trocar in the umbilicus. In addition, two further 5-mm trocars were positioned along the right mid-abdomen. Through one of these, the surgeon employed a grasper with the left hand to secure the gallbladder infundibulum in the left mid-abdomen, while the other one enabled the surgeon to manipulate a dissector with the right hand. Moreover, one more 12-mm trocar was placed beneath the xiphoid to permit upward retraction of the gallbladder fundus across the liver. The initial steps of the dissection were performed by the assistant, who retracted the fundus of the gallbladder across the surface of the liver, while lateral traction of the gallbladder infundibulum was applied by the surgeon’s one hand. During dissecting for a critical view of safety in Calot’s triangle, an unexpected anatomical pulsatile structure was revealed entering the hepatocystic triangle (Figure 1). Dissection was performed to separate the visceral peritoneal layer at the gallbladder neck. During further meticulous assessment, the right hepatic artery was recognized entering the Calot’s triangle with a tortuous course above the cystic duct. Identification of the cystic artery and its anterior and posterior branches, together with the cystic duct and the twisting vessel, facilitated access to the critical safety triangle. The borders of this space were defined by the gallbladder wall on the right, the cystic artery on the left, and the cystic duct inferiorly. Through this triangle scheme, the liver surface could be observed, superior to Rouviere’s sulcus, following the description by Strasberg et al. [7,8]. The cystic duct and artery were clipped at both ends with Hem-o-lok® (Teleflex, Morrisville, NC, USA) and then divided after careful identification. When a tortuous right hepatic artery formed a prominent curve adjacent to the gallbladder and cystic duct, the short cystic artery was clipped and divided, allowing safe separation of the hepatic artery from the gallbladder (Figure 2). A hook instrument was employed to separate the gallbladder from the hepatic bed, followed by its enclosure in an Endo Catch™ bag (Covidien, Mansfield, MA, USA) and its extraction through the 12-mm trocar site. Following this procedure, a subhepatic drain was placed and maintained for 24 hours. The patient recovered without complications and was discharged on the first postoperative day. Informed consent was obtained from the patient.

## Discussion

Theories of pathogenesis

Many theories have been proposed regarding the formation of Moynihan’s hump. Specifically, Taylor argued that cirrhosis of the liver causes significant deformation of the internal architecture, creating a twisting right hepatic artery [9]. Moreover, other theories support the idea that traction during cholecystectomy created an artifact and not an anomaly. However, both of the aforementioned ideas have been disproven due to a lack of evidence or contradicting proof [10]. Finally, Miyaki presented his belief that Moynihan’s hump was “established” by embryological causes. Precisely, it is known that three arteries, all arising from the aorta, provide blood to the liver during embryonic development. Later, these three arteries give rise to the hepatic artery as well as accessory hepatic arteries originating from the left gastric and superior mesenteric arteries. Given that the branch from the left gastric artery persists in about 25% of individuals and the branch from the superior mesenteric artery in 18.3%, it has been suggested that the right hepatic artery’s Moynihan’s hump may result from partial or complete persistence of fetal arterial branches [9,11].

Thus, the present case report aims to describe an intraoperatively identified Moynihan’s hump of the right hepatic artery encountered during laparoscopic cholecystectomy. It emphasizes the surgical significance of this variation, the technical considerations for safe dissection, and the importance of recognizing it to prevent iatrogenic vascular injury.

Anatomical types of Moynihan’s hump

To better understand the anatomical formation and the location of Moynihan’s hump, its location is described in relation to the cystic duct. Therefore, this variation can be classified as supracystic if the hump is observed anterior or posterior to the common hepatic duct, superior to the cystic duct, and nearer to the hilum of the liver. A paracystic hump refers to its location around the convergence of the cystic artery with the common hepatic duct. In this category, high chances of structural damage are reported due to the proximity of the cystic artery and Moynihan’s hump. Finally, an infracystic hump is characterized as such when the hump is observed below the aforementioned convergence, closer to the duodenum [12].

**Figure 3:**
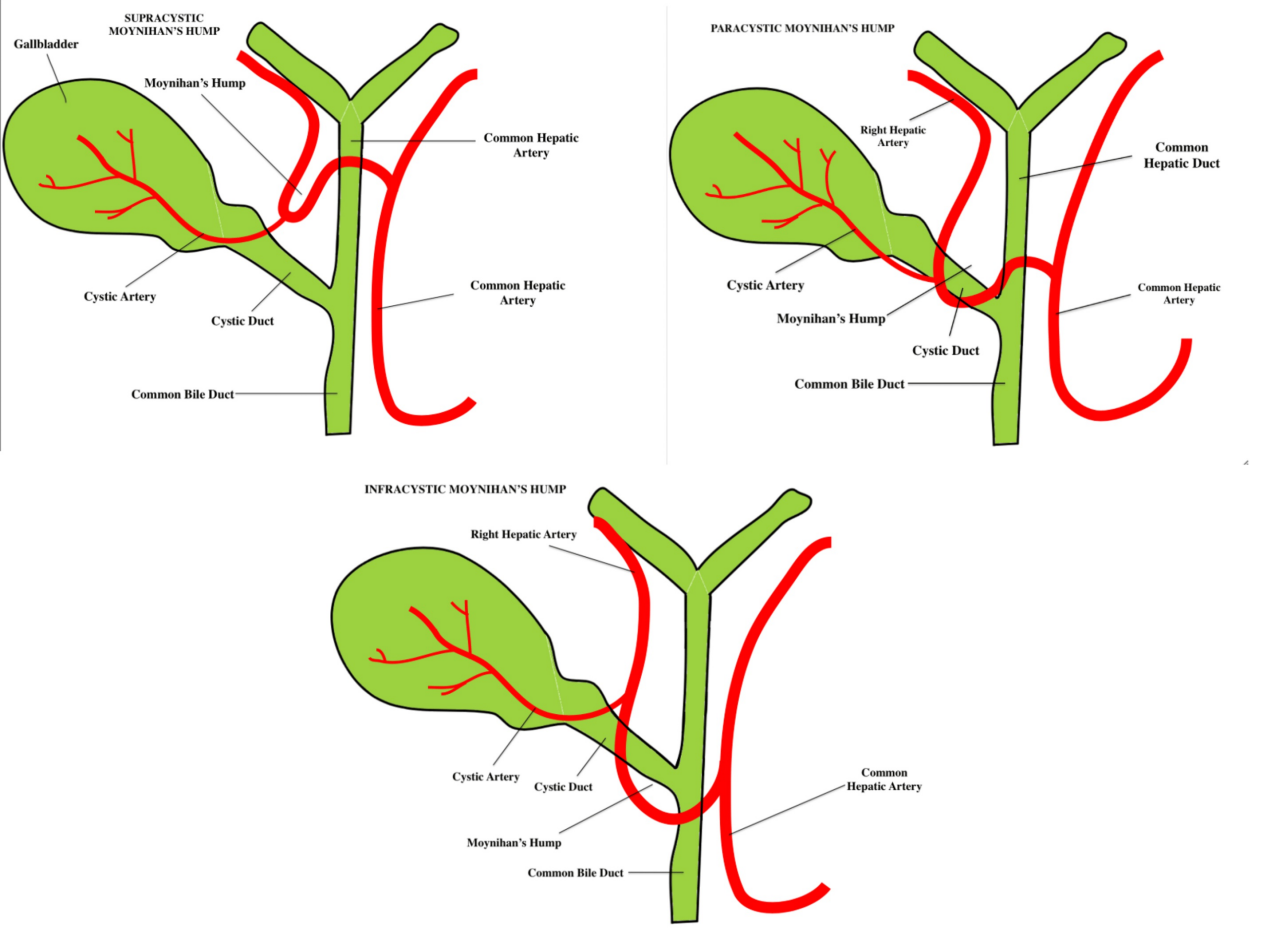
Anatomical types of Moynihan’s hump. Top left: Supracystic Moynihan’s hump. Top right: Infracystic Moynihan’s hump. Bottom: Paracystic Moynihan’s hump.

**Figure 4:**
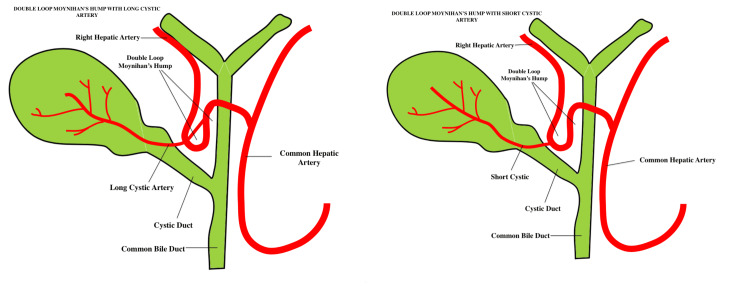
Anatomical classifications of Moynihan’s hump. Left: Double-loop Moynihan’s hump with a long cystic artery. Right: Double-loop Moynihan’s hump with a short cystic artery.

Another way to characterize and categorize the different types of Moynihan’s hump is according to the presence of a single or a double loop. Precisely, the twisting right hepatic artery can create a single or a double loop that runs either anteriorly or posteriorly to the common hepatic duct. In double-loop variants, the cystic artery typically originates from the adjacent segment and traverses the right hepatic artery en route to the gallbladder. In other cases, the double-loop artery can be reported to emerge distally and be notably short in length [6,13]. In our case, the tortuous hepatic artery was classified as a supracystic hump with a single-loop configuration, while also being accompanied by a short cystic artery. Additionally, the artery of our patient was observed to pass dorsally to the common hepatic duct, requiring careful assessment. The different anatomical types of Moynihan’s hump are depicted in Figure 3 and Figure 4.

Possible complications

This anatomical variation holds significant surgical gravity, as the twisting structure of the right hepatic artery may become the target of a serious iatrogenic injury if it is misidentified as the cystic artery. In general, this medical error can provoke multiple severe complications, including hepatic ischemia, necrosis, atrophy, or abscess formation [14,15]. Specifically, the gravity of injury of the right hepatic artery dictates the extent of the structural damage. In cases of complete ligation, ischemic necrosis of the right liver lobe may be reported. Moreover, partial injury is capable of causing hepatic artery pseudoaneurysm and life-threatening bleeding. Finally, any form of vascular hemorrhage can hinder the surgeon’s field of vision, which leaves the patient vulnerable to unwanted injury due to “blind” clipping and coagulation [13]. As the abnormal right hepatic artery gives a relatively short cystic artery, any excessive traction applied to the gallbladder may lead to its extrication from the hepatic artery, causing severe bleeding [12,16,17]. In our case, the significance of preventing such injury was demonstrated, as the patient’s short cystic artery necessitated careful and coordinated gallbladder retraction by the surgeon and assistant. However, there are cases where the “twisting” hepatic artery does not give just one short cystic artery but several small ones that supply the gallbladder. Therefore, unwanted injuries may be observed even during the physician’s attempt to secure them. The fact that the right hepatic artery supplies blood to the bile duct may result in bile duct narrowing secondary to ischemia [6]. Thus, the patient may present with cholangitis and cirrhosis.

Prevention of structural damage

To prevent such an injury, the presence of Moynihan’s hump should be suspected when a cystic artery of unusually large caliber is observed. It is significant to remember that the diameter of the blood vessels is not a reliable and definitive method for differentiating the cystic artery from the right hepatic artery [12]. Thus, ensuring clear visualization of the right hepatic artery around the cystic artery origin is fundamental. In other words, medical professionals are advised to identify the variation, dissect it, and locate the origin of the cystic artery. Visualization of the area of interest is achieved through the lateral retraction and dissection of the infundibulum of the gallbladder to isolate the cystic artery termination [2,4]. The importance of this maneuver is highlighted by its role as an initial step in our surgical strategy. Another possible practice used by medical physicians to ensure a low risk of structural damage is the use of intraoperative cholangiography [2,18]. According to Strasberg et al., it is advised that no invasive procedure (division or clipping) should be performed unless professionals have examined Calot’s triangle and identified two crucial features, the cystic artery and the cystic duct [2,10]. For this purpose, in 1995 and 2010, Strasberg et al. described a strategy to avoid any biliary duct injuries, which was named “critical view of safety.” This strategy highlighted the importance of identifying the cystic structures before their dissection. Thus, Calot’s triangle would be cleared of fat and fibrous tissue, followed by the partial dissection of the gallbladder off the cystic plate. Following these steps, the visualization of the cystic artery and cystic duct was achieved [2,10,11]. Strasberg’s instruction regarding the visualization and the identification of the crucial contents of Calot’s triangle was showcased in this case. Although no intraoperative cholangiography was performed, surgical vigilance, along with meticulous adherence to the existing instructions, ensured a safe and successful procedure.

Some professionals suggest that in the case of injury of the right hepatic artery during a laparoscopic cholecystectomy, a direct reconstruction with an end-to-end anastomosis is preferable, as it minimizes the risk of potential complications. On the other hand, some advise against any repair, as it is possible for a patient with a healthy, normal liver to be asymptomatic [6,19,20].

## Conclusions

Moynihan’s hump is a rare tortuous variant of the right hepatic artery that may course within Calot’s triangle. In this case, the anomaly was identified intraoperatively and managed safely with meticulous dissection, preventing vascular or biliary injury. Misidentification of this vessel can result in severe complications, emphasizing the importance of its anatomical awareness. Careful dissection, minimal traction, and precise identification of cystic structures are essential to ensure operative safety and optimal outcomes.
